# Genome Editing in Plants: Exploration of Technological Advancements and Challenges

**DOI:** 10.3390/cells8111386

**Published:** 2019-11-04

**Authors:** Sanskriti Vats, Surbhi Kumawat, Virender Kumar, Gunvant B. Patil, Trupti Joshi, Humira Sonah, Tilak Raj Sharma, Rupesh Deshmukh

**Affiliations:** 1 National Agri-Food Biotechnology Institute (NABI), Mohali Punjab 140306, India; Sansvats@gmail.com (S.V.); Surbhikumawat002@gmail.com (S.K.); vvs.kumar15@gmail.com (V.K.); 2 Department of Agronomy and Plant Genetics University of Minnesota, St. Paul, MN 55108-6026, USA; Gunvantpatil@gmail.com; 3 Department of Health Management and Informatics; Informatics Institute; Christopher S Bond Life Science Center, University of Missouri, Columbia, MO 65211-7310, USA; joshitr@health.missouri.edu

**Keywords:** CRISPR/Cas, multi-target editing, promoter bashing, methylome-editing, biotic and abiotic stress tolerance, plant transformation

## Abstract

Genome-editing, a recent technological advancement in the field of life sciences, is one of the great examples of techniques used to explore the understanding of the biological phenomenon. Besides having different site-directed nucleases for genome editing over a decade ago, the CRISPR/Cas (clustered regularly interspaced short palindromic repeats/CRISPR-associated protein) based genome editing approach has become a choice of technique due to its simplicity, ease of access, cost, and flexibility. In the present review, several CRISPR/Cas based approaches have been discussed, considering recent advances and challenges to implicate those in the crop improvement programs. Successful examples where CRISPR/Cas approach has been used to improve the biotic and abiotic stress tolerance, and traits related to yield and plant architecture have been discussed. The review highlights the challenges to implement the genome editing in polyploid crop plants like wheat, canola, and sugarcane. Challenges for plants difficult to transform and germline-specific gene expression have been discussed. We have also discussed the notable progress with multi-target editing approaches based on polycistronic tRNA processing, Csy4 endoribonuclease, intron processing, and Drosha ribonuclease. Potential to edit multiple targets simultaneously makes it possible to take up more challenging tasks required to engineer desired crop plants. Similarly, advances like precision gene editing, promoter bashing, and methylome-editing will also be discussed. The present review also provides a catalog of available computational tools and servers facilitating designing of guide-RNA targets, construct designs, and data analysis. The information provided here will be useful for the efficient exploration of technological advances in genome editing field for the crop improvement programs.

## 1. Introduction

Cells have several inherent mechanisms for the repair of double-strand DNA breaks (DSBs) [1,2]. These DNA repair mechanisms have been acknowledged as important approaches for targeted gene modification or editing, by introducing precise breaks in the genome at specific sites. Earlier approaches to modify the genomic DNA and RNA included self-splicing introns, cross-linking agents like psoralen or bleomycin or other chemical reagents coupled with chemical recognition of DNA sequences using polyamides or peptide nucleic acids (PNAs), and homing endonucleases encoded by introns [3,4,5,6,7]. These strategies relied on the Watson–Crick base pairing in the nucleic acids. In 1994, Rouet and Smith for the first time performed experiments with rare-cutting meganuclease, I-SecI which showed that introduction of intentional DSBs using meganucleases could be used to achieve local mutagenesis as well as the incorporation of homologous donor sequences at the target genomic site [8]. Since then, the field of genome editing has forged ahead enormously.

The current advancements for editing genes include site-specific nucleases, usage of which for genome editing began with the advent of zinc-finger nucleases (ZFNs) in 2002. The ZFNs were the first truly target specific protein reagents that revolutionized the field of genome manipulation. ZFNs are DNA binding domains and specifically recognize three base pairs at the target site [9]. Two DNA-binding ZFNs are attached with *FokI* monomer and constructed to create a spacer of 5–6 bp, enabling the *FokI* to be functional on dimerization and create DSBs. [10,11]. The fusion protein of zinc fingers with *Fok*I nuclease had already been established as a restriction enzyme in 1996 by Kim et al. [12], but their use as site-specific editing tool began only after 2002 when Bibikova and associates used ZFNs to induce mutagenesis and targeted chromosomal cleavage in *Drosophila* [11]. It involves the introduction of targeted DSBs using ZFN that stimulates cellular DNA repair mechanisms. Since then, ZFNs have been used widely for targeted genome modifications in various plant species such as *Arabidopsis* [13,14], tobacco [15,16,17], and maize [18]. The second class of site-directed mutagenesis, transcription activator-like effector nucleases (TALENs), identified first in plant pathogenic bacteria (*Xanthomonas)*, function on a similar principle as ZFNs. TALENs target one nucleotide (instead of three) at the target site, making TALENs highly specific [19,20]. TALENs have successfully been used to perform genome editing in angiosperms as well as bryophytes [21,22,23]. However, difficulties of protein synthesis, design, and validation needed for TALENs and ZFNs are some of the constraints for the widespread implementation of these nucleases for regular use.

**Figure 1:**
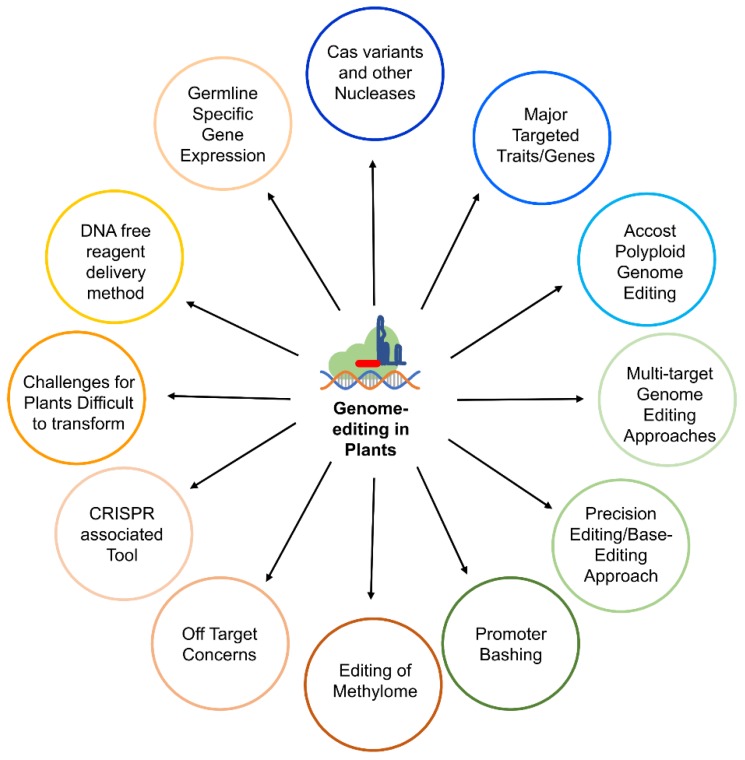
An overview of the different aspects covered in the present review related to the CRISPR/Cas (clustered regularly interspaced short palindromic repeats/CRISPR-associated protein) based genome editing in plants.

**Figure 2:**
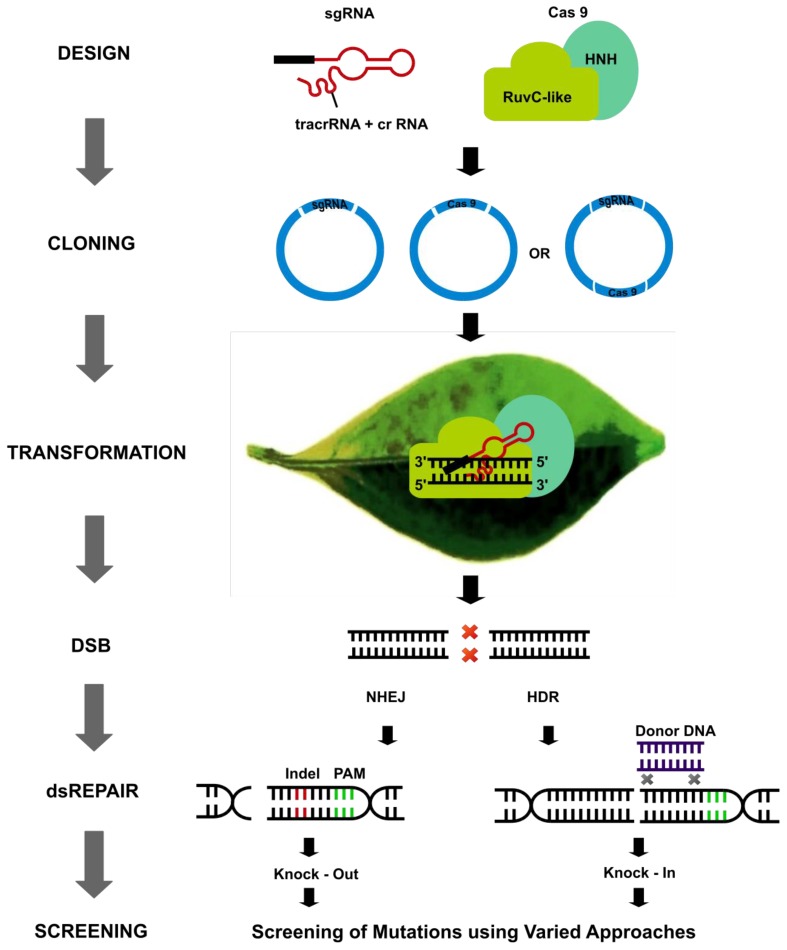
Generalized process for CRISPR/Cas mediated genome editing in plants. sgRNA is composed of a spacer (black), and crRNA and tracrRNA (both shown here in red), and Cas9 is composed of two domains: HNH and RuvC-like domain. HNH domain cleaves the DNA strand complementary to the sgRNA, and RuvC-like domain cleaves the other DNA strand. Cas and sgRNA coding sequences are cloned into a vector (blue), together or individually, which is transformed into the plant cells. The sgRNA and Cas9 are expressed in the plant which then leads to double-strand break (DSB), resulting in activation of DNA repair machinery leading to the modification of DNA sequence and subsequently in the protein coded by sequences and conclusively in the phenotype. The final step is the screening of mutations, which is usually done by PCR and sequencing. Abbreviations: Cas9: CRISPR associated protein 9; crRNA: CRISPR RNA; DSB: double-stranded break; dsREPAIR: double-strand repair; HDR: homology directed repair; Indel: insertion or deletion mutations; NHEJ: non-homologous end joining; sgRNA: single guide RNA; tracrRNA: transactivating CRISPR RNA.

Recently developed CRISPR/Cas (clustered regularly interspaced short palindromic repeats/CRISPR-associated protein) technique based on type II prokaryotic adaptive immune system, that helps bacteria or archaea against the invading phages, provides an excellent alternative to the first generation site-directed nucleases [24,25]. TALENs and ZFNs were successfully used for gene editing, but CRISPR/Cas provides several advantages in terms of design, specificity, multiplexing, cost, and flexibility over other methods. Recent efforts have expanded the utility of CRIPR/Cas system by exploring various fundamental aspects of the biological mechanisms (Figure 1). CRISPRs were identified in bacterial DNA as early as 1987 [26], but their function in bacteria was not understood until 2005 [27,28], and in 2007 CRISPRs were proved to provide immunity in combination with Cas protein [29]. In 2012, Doudna and associates were the first ones to perform gene editing using CRISPR/Cas in a cell-free system [24], and shortly afterward five independent groups applied this system for editing genes in the animal system [25,30,31,32,33]. First reports of gene editing in plants using CRISPR/Cas9 came in 2013 in the model plants Arabidopsis and *Nicotiana benthamina* [34,35] and rice [36]. Three types of CRISPR/Cas systems, utilizing different molecular mechanisms are found in bacteria. The CRISPR/Cas system used was derived from the prokaryotic type II CRISPR system of *Streptococcus pyogenes*, which includes precursor CRISPR RNA (pre-crRNA), trans-activating crRNA (tracrRNA) and Cas9 nuclease. The tracrRNA is involved in the maturation of pre-crRNAs into crRNAs [37]. Type II system utilizes a single protein for target recognition and cleavage. Dual tracrRNA:crRNAs are engineered as single guide RNAs (sgRNAs) while using CRISPR/Cas for genome editing. sgRNAs retain two critical features, 5′ sequence that is complementary to the target DNA and 3′ sequence that binds to the Cas9 protein. DSBs generated can be repaired by non-homologous end joining (NHEJ) and homology directed repair (HDR) to create gene knockout or gene knock-in type of modifications, respectively (Figure 2) [38]. NHEJ is the most dominating and active pathway in eukaryotes for repairing DSBs, leading to small insertions or deletions (indels), thus producing a gene knockout or silencing of a gene. HDR can be utilized in the presence of a repair template, and can result in insertion, translocation, or inversion of gene sequences. HDR can lead to knock-in, protein-domain swaps, new gene functions or alteration in gene regulation. HDR can induce more desirable mutations, but the preference of NHEJ over HDR in natural systems prevents its efficient and more frequent application in practical systems. Since its inception, CRISPR/Cas has progressed rapidly and has been applied successfully in a wide range of plants and on varied traits (Table 1). This review deals with such technological advancements, including various multi-targeting approaches, precision editing, epigenome editing, and various other aspects.

## 2. Cas Variants and Other Nucleases for Plant Genome Editing

Cas9 is a DNA specific endonuclease that is found in bacterial species—such as *Streptococcus pyogenes*, *Streptococcus aureus*, *Streptococcus thermophilus*, *Francisella novocida*, and *Brevibacillus laterosporus*—out of which the *Streptococcus pyogenes* Cas9 (SpCas9) is predominantly used. Cas9 is a multifunctional protein having two nuclease domains, HNH and RuvC-like domain. Cas9 can be modified into a nickase, capable of producing a single strand cleavage, by mutating either the HNH or the RuvC-like domain. Similarly, Cas9 can also be customized into a DNA binding protein, dead Cas9 (dCas9) by mutating both the domains (dCas9; Asp^10^→Ala, His^840^→Ala). The SpCas9 uses a 5′-NGG-3′ protospacer adjacent motif (PAM), and even though 5′-NGG-3′ sequence occurs approximately 5–10 times in every 100 bp in model plant species [47] the PAM requirement is still a bottleneck for the Cas9 targetable sites. To overcome this issue, many Cas9 variants and Cas9 orthologs with various PAM preferences have been used to achieve the same results as the wild type CRISPR/Cas9 system. One of such systems is CRISPR from Prevotella and Francisella (Cpf1), which is more recently cited as Cas12a, is a nuclease of class II type V and lacks the HNH domain, possessing only the RuvC-like domain naturally. Cpf1 yields break sites with staggered cuts rather than blunt ends as Cas9 [48]. Cpf1 requires a T rich PAM that increases the number of possible plant genetic manipulations and a shorter crRNA than Cas9 [49]. However, short crRNAs increase the possibility of having a secondary structure in the RNA. Also, Cpf1 edited lines may need precise genomic evaluation as Cpf1 has been shown to cause genomic rearrangements in regions surrounding the target sites [50]. Nonetheless, Cpf1 has already been used in many plant species such as rice [43,51,52] and Arabidopsis [53] and offers a great alternative to Cas9 and a wider range of targetable genes in addition to the ones offered by Cas9. More recently, a new class II system encoding a miniature (529 amino acids) effector, Cas14a1, has been identified [54] Importantly, this Cas variant functions as a PAM-independent single stranded DNA nuclease. Many more Cas variants and orthologs are being discovered [55] and exploited for gene editing purposes since the CRISPR/Cas system is a general immune system present in bacteria and archaea for protection against bacteriophages.

## 3. Genes Targeted for Genome Editing in Plants

Genome editing has also been used in plants for functional annotation of various genes previously deciphered and proved to be associated with many vital processes. For example, stress-related genes, ideal marker genes, genes related to plant architecture have been targeted, which are discussed in the subsequent sections.

### 3.1. Evaluation of CRISPR/Cas Efficiency Using Easily Scorable Marker Genes

CRISPR/Cas9 is still in preliminary stages of development, therefore the most frequently targeted genes used for genome editing includes the genes which have been proved to be an easily scorable marker for plants (Table 1). Genes regulating pigmentation in plants have been used most frequently to evaluate the precision and efficiency of the CRISPR/Cas9 system. One such gene is *Phytoene desaturase (Pds)*, which functions in the carotenoid synthesis pathway and converts 15-cis phytoene to zeta-carotene. The knockout of this gene results in decreased carotenoid synthesis, thereby leading to photobleached or albino phenotype. Most of the preliminary work have targeted *Pds* gene to demonstrate the successful application of CRISPR/Cas9 in different plant species [34,39,41,56], including horticultural woody plants, such as *Actinia chinensis* (kiwifruit) [57], *Coffea canephora* (coffee) [58], *Mannihot esculenta* (cassava) [59], and apple [60]. Many other marker genes frequently used to study CRISPR/Cas9 include *Chloroplastos alterdos (Cla1)*, *Coumarate;CoA ligase (CL)*, *Rice Outermost Cell-specific (ROC)*, *Brassinosteroid insensitive (Bri), Gibberellic acid insensitive (Gai)*, and *Young seedling albino (Ysa).* Mutations in these genes produce easy to score phenotypes, like *Cla1*, which is involved in chloroplast development and when silenced results in an albino phenotype in the true leaves of cotton [61,62]. Similarly, the silencing of *ROC* results in curly leaf phenotype in rice plants [40]. However, these genes can interfere with normal growth and development of seedlings, as is characterized in case of *Pds.* Mutation in *Pds* results in stunted growth due to insufficient nutrition availability, a result of abnormal chloroplast development in the proplastid stage. Therefore, marker genes like *Ysa* should be preferred as it produces albino phenotype only in the seedling stage and has no negative effect on other agronomical traits at the later stages of plant growth. Thus, *Ysa* gene has been effectively used in rice for genome editing, indicating successful alterations in the genetic material without interfering much with plant growth and. Although the exogenous application of Gibberellic acid (GA) has been proved to help rectify the alterations produced by *Pds3* in *Arabidopsis* [63]. Such factors can be identified and used in the culture to correct the negative alterations produced by marker genes. Additional marker genes frequently used for genome editing are β-glucuronidase (GUS) and green fluorescent protein (GFP), but these genes cannot be used efficiently since the GUS requires histochemical assaying for detection and GFP requires special methods to detect the inflorescence and differentiate inflorescence from normal colors. Therefore, proper selection of marker genes is required for efficient evaluation of genome editing tools.

### 3.2. Translational Efforts by Targeting Genes Previously Annotated with RNAi

Genome editing is currently being applied to create a knockout mutation in numerous genes previously annotated by using RNAi. For instance, CRISPR/Cas9 approach has been used to knockout *Mildew Locus O* (*MLO-7)* in grapevine [64], *Self-pruning 5G* (*SP5G)* in tomato [65], and *ROC gene5* in rice [40] which were previously characterized by RNAi technology. Targeting previously annotated genes is indispensable for two major reasons, firstly CRISPR/Cas is still at preliminary stages of its application that need conformational studies, and secondly to circumvent the stringent and costly regulations raised for the commercial release of transgenic varieties developed using RNAi technology. Crops improved using genome-editing, without any foreign piece of DNA, have already been given the non-transgenic tag by the United States government and the same is being anticipated from other countries as well. Therefore, for tranquil, inexpensive, profitable, and commercially facile release of improved crop varieties, gene editing using the CRISPR/Cas system is being preferred over RNAi. Another factor is the possibility of targeting multiple genes simultaneously using CRISPR/Cas. Genes earlier annotated using RNAi have been included in multi targeting constructs of CRISPR/Cas, such as *pectate lyase (PL)*, which is involved in tomato fruit softening [66]. *PL* was targeted along with two other pectin degrading enzymes, namely polygalacturonase 2a (PG2a) and β-galactanase (TBG4) for the comparative analysis of tomato cell wall mutants [67]. Analyzing mutants in different experimental systems is quite difficult and ambiguous. CRISPR/Cas provides an opportunity to mitigate this difficulty by targeting multiple genes at once. Multiplexed gene targeting is discussed later in this review in detail.

### 3.3. Genes for the Enhancement of Resistance against Biotic Stresses

Significant yield losses occur worldwide every year because of biotic and abiotic stresses. To achieve sustainable and secure yield enhancement, engineering crop plants against stress conditions is a valuable task. Biotic stresses faced by plants include fungal pathogens, bacteria, nematodes, plant parasites, harmful insects, and plant viruses. Plant viruses alone cause damage of up to 10–15% in crop yield globally [68]. Ali et al., (2016) have demonstrated efficient targeting and cleavage of *Cotton leaf curl Kokhran virus* (CLCuKoV) and also illustrated that simultaneous resistance for multiple begomoviruses can be developed by targeting the conserved nonanucleotide sequence (TYLCV, CLCuKoV, TYLCSV, BCTV-Worland, MeMV, and BCTV-Logan), thus conferring broad-spectrum geminivirus resistance to *N. benthamiana* plants [69]. A significant factor to be addressed is the ability of geminiviruses to evade the CRISPR/Cas9 reagents. This can be more frequent due to the continuous arms race between the host plants and invading viruses. Reports suggest that targeting coding sequences lead to viral variants capable of evading the CRISPR/Cas9 system, and in contrast to this, elevated viral interference, and no viral escapes from the CRISPR/Cas9 system were uncovered when non-coding intergenic sequences were targeted [70]. Recently, Tashkandi et al. (2018) have also developed tomato plants resistant to TYLCV, a begomovirus, by targeting the coat protein (CP) or replicase (Rep) with single nucleotide changes being the predominant modification observed in Cas9 targeted sites [71].

Targeting RNA viruses with CRISPR/Cas9 reagents has been a challenge since the default target for sgRNA-Cas9 is DNA. However, there is still a possibility as the Cas9 can be programmed to target RNA [72], and Type III-B and type VI-A CRISPR/Cas system from *Leptotrichia shahii* (LshCas13a) or *Leptotrichia wadei* (LwaCas13a) also mediate cleavage of RNA sequences complementary to the sgRNA [73]. Recently, Zhang et al. (2018), have accomplished successful application of the above said concept by programming sgRNA specific for the RNA genome of cucumber mosaic virus (CMV) or tobacco mosaic virus (TMV). Using *Francisella novicida* Cas9 (FnCas9) [74], they engineered *Nicotiana benthamiana* and *Arabidopsis* plants with effectively reduced viral titer. Alternate strategies which have been developed to mitigate this problem include targeting plant gene that is directly involved in infection, instead of the viral RNA, to render the plant resistant to the viruses. Initiation factors like *eIF4E* and *eIF(iso)4E* are one of such genes, which had already been proved in *Arabidopsis* to be involved in *Turnip mosaic virus* (TuMV) infection. The CRISPR/Cas9 system has been successfully used to develop *Arabidopsis* plants resistant to the potyvirus TuMV using this approach [75]. Cucumber (*Cucumis sativus* L.) plants partially resistant to two potyviruses namely *Papaya ringspot mosaic virus*-W and *Zucchini yellow mosaic virus* and an ipomovirus like *Cucumber vein yellowing virus* have also been developed by targeting *eIF4E* [76]. Disrupting *eIF4E* and *eIF(iso)4E*-like host factors have certain supplementary benefits, as numerous natural foundations of *Potyvirus* resistance arise from loss-of-function mutations in the host initiation factors [77], therefore, providing broad-spectrum resistance. Although the translation initiation factors are key candidates that can be targeted in the host genome [77], any of the host genes that encodes a factor required by a virus for the effective spread of infection, is a conceivable target for alteration. The effective use of CRISPR/Cas9 to develop resistance against viruses, specifies the potential significance of this system to manage viral diseases in crops.

Gene editing has also been successfully used to improve plant resistance against fungal and bacterial pathogens [78]. Significant efforts have been made towards powdery mildew resistance in several crop species. The use of fungicides can efficiently control powdery mildew, but the rapid evolution of fungal strains to develop resistance to these fungicides and the additional costs to growers, together with the hazardous effect of fungicides on the environment necessitates the development of alternative strategies. The most common practice in developing resistant varieties is targeting susceptible genes (S gene), *MLO*, which suppress the defense system of plants against powdery mildew [79]. This denotes that loss-of-function mutations in the *MLO* alleles should lead to broad-spectrum resistance to the powdery mildew. Powdery mildew in wheat is caused by *Blumeria graminis f. sp. tritici (Bgt)*, one of the most damaging plant pathogens in wheat production. Wang et al. (2014) have successfully knocked out all of the homologs of the *MLO* gene in hexaploid wheat by deploying gene editing approach, which renders durable resistance to *Bgt* in wheat plants [79]. In another study, CRISPR/Cas9 edited tomato plants named as “tomelo” were developed to confer resistance against powdery mildew by targeting *MLO* genes [80]. Similarly, the susceptibility (S) gene, *MLO7* has also been targeted in grape for controlling *Erysiphe necator* infection, a fungal agent that causes powdery mildew in grapes [64]. Here, ribonucleoproteins (RNPs) were used to directly deliver CRISPR/Cas9 reagents to the protoplasts of grape cultivar *Chardonnay*. A similar approach was used in the same study, for developing apple plants resistant to fire blight pathogen, *Erwinia amylovora*, which is an enterobacterial phytopathogen. For this purpose, *DIPM-1, DIPM-2*, and *DIPM-4* genes were targeted for genome editing [81]. CRISPR/Cas9 system for genome editing has also been successfully exploited in developing resistance to blast disease in japonica rice by targeting codons close to translation initiation codon of OsERF922 with a sgRNA to introduce indels [82]. These mutant lines further characterized for many agronomic traits including flag leaf width, flag leaf length, plant height, number of panicles, rate of seed setting, length of panicle, and seed weight, and none of the observed traits significantly differed from wild-type plants, implying that alteration of OsERF922 can yield plants with increased resistance without any negative effect on plant development. Genome editing approaches seem promising for combating devastating diseases in crop plants

### 3.4. Genes for the Enhancement of Abiotic Stress Tolerance in Plants

Abiotic stresses are the most serious constraints in agricultural production, and the negative impact is bound to worsen with global climate change. Abiotic stress tolerance is governed by a number of genes and largely influenced by environmental factor which make it challenging to study [83,84]. Conventional breeding techniques and transgene-based systems, although have helped to develop resilient crop varieties, but the complex inheritance of abiotic stress-related traits and higher environment effects make it very difficult to develop novel cultivars using conventional methods. Similarly, induced mutagenesis is a widely explored method for the genetic improvement of several crop species besides being entirely dependent on random events [85,86]. CRISPR/Cas9 system can be utilized for forward genetics where manipulation of genes and gene expression can be performed to study the genetics of abiotic stress response, and thus assist in producing the stress-resistant crop varieties. The CRISPR/Cas9 approach, which is now being largely exploited in plant science, is restricted to a very few publications related to its application for the understanding and development of abiotic stress-resistant plants. Shi et al. (2017) have developed a corn variety through CRISPR/Cas based genome editing approach which has improved yield under drought stress. The study has targeted ARGOS8 that negatively regulates ethylene responses. Improved expression of ARGOS8 in genome-edited plants showed enhanced drought tolerance [87]. In another study, a tissue-specific AtEF1 promoter was used to drive truncated gRNAs (tru-gRNAs) and *Cas9*, which caused mutations in abiotic stress-responsive genes, namely *OST2/AHA1* [88], leading to enhanced stomatal responses in *Arabidopsis*. Rice genes OsRR22 and OsNAC041 have also been targeted to increase salinity tolerance [89,90]. A recent study has been successful in targeting 25 different genomic targets by leveraging RNase/DNase property of *Acidaminococcus* Cas12a (Cpf1) for multiplexed genome editing [91]. The approach mentioned above can be helpful for simultaneously targeting the multiple genes involved in abiotic stress. Aquaporins are some of the prime candidates for abiotic stress enhancement, where genome editing can be employed to modulate solute transport regulations, particularly water, urea, H_2_O_2_, and silicon [92,93,94]. Similarly, other transporter proteins are also prominent candidates for genome editing for the enhancement of abiotic stress tolerance [95,96,97]. These factors indicate that the CRISPR/Cas system can be harnessed prolifically for this novel purpose and will be the future of targeting minor genes of complex quantitative traits related to abiotic stresses [98].

## 4. Editing Polyploidy Genomes—Challenges and Perspective

The introduction of desirable traits in leading crop varieties using classical breeding approaches is a very challenging and time-consuming task when it comes to very complex polyploid genomes such as sugarcane, cotton, wheat, and potato. Introgression of multiple traits and modification of metabolic pathways is also tricky with conventional breeding approaches in polyploidy plants. However, genome editing techniques offer several advantages over the conventional breeding process, where multiple genes or metabolic pathways can be targeted at the same time, without any linkage drag. CRISPR/Cas9 can especially be very efficiently used for many purposes and has already been exploited to generate broad-spectrum resistance to powdery mildew in wheat [79], and has also been used to generate mutations in cotton [56,61,99,100,101,102], Duncan grapefruit [103,104], and potato [105,106,107,108]. Nevertheless, the large genome size and high copy number in polyploidy crops possess several challenges in site-directed mutagenesis. These challenges include knock-out of multiple genes with high homology, but it can be overcome by generating a series of allelic variants and segregating them in the next generation to select desirable genotype.

Sugarcane being a polyploid, is a classic case of complexities for the efficient exploration of gene editing techniques. Simultaneous manipulation of all the homologs in sugarcane looks challenging, primarily due to its large genome size (10 GB) and the number of homologous copies of genes ranging from about 8 to 10 [109]. In sugarcane, the chromosome number varies even among species of the same genus, and the species are also interfertile [110]. Transgene silencing in sugarcane, which is independent of copy number of genes, very common in primary transformants, primarily post-transcriptional in T0 plants, and regulated by the stage of development of plant [111], is a major issue that obstructs the successful application of CRISPR-Cas. Birch et al. (2010), have shown that this transgene-silencing is promotor sequence-specific, which makes it obligatory to choose diverse and proficient promoters to trim down the silencing effects [111]. Lack of an annotated genome for sugarcane also makes it tough to design sgRNAs to target genes in this crop.

Another fundamental limitation is the requirement of a large number of mutants to study the multiple forms of alleles present in polyploids. Multiple sgRNAs can achieve mutagenesis in crops with annotated genome like wheat and cotton, but with sugarcane, even this seems complicated [112]. Nevertheless, with advancing technologies and handy bioinformatics tools, CRISPR-Cas will be the most widely used tool for molecular improvement of polyploids in the near future.

## 5. Multi-Targeting Genome Editing Approaches

One of the major and prevalent advantages of CRISPR/Cas9 technology is that it can be used to target multiple genes (or multiple sites within a gene) to create small or large deletions in the genome and provides practical applications in basic and applied biological research. In general, two approaches have been used for expressing multiple gRNA. In the first approach, each gRNA is expressed with an individual promoter and in second approach multiple gRNAs expressed by one promoter as a single transcript which is further processed or cleaved off to release individual gRNAs [113]. Currently, there are several efficient strategies developed to achieve CRISPR/Cas9 enabled multiplex genome editing, which are discussed below.

### 5.1. t-RNA Mediated Multi-Targeting Genome Editing

**Figure 3:**
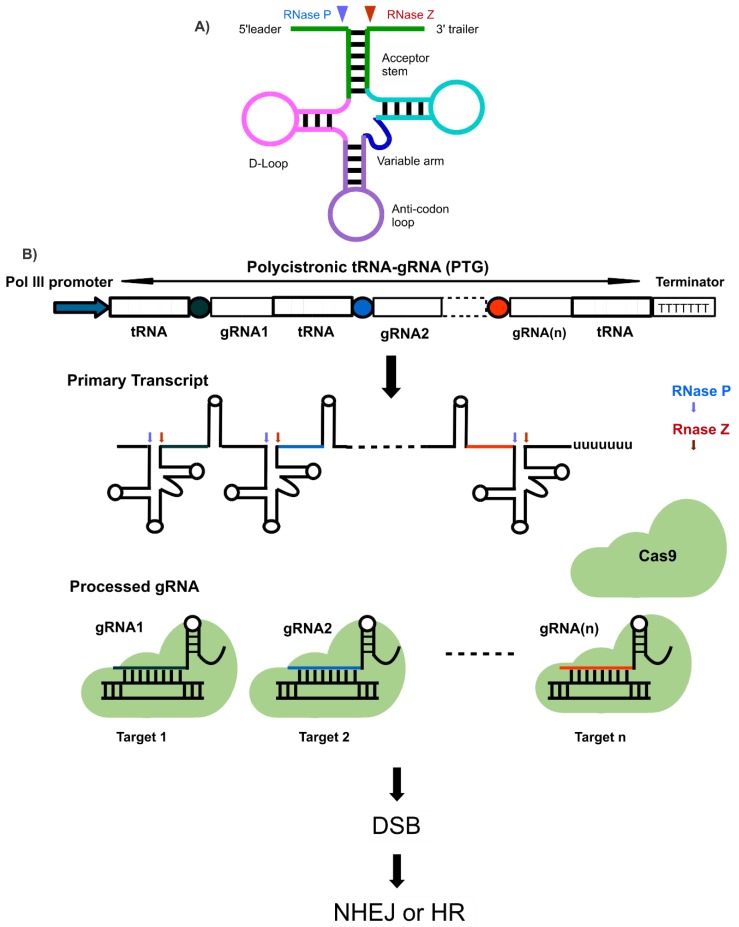
Multigene targeting via CRISPR/Cas9 using PTG/Cas9 method. (**A**) A eukaryotic pre-tRNA with a depiction of post-transcriptional processing by RNaseP and RNaseZ (depicted as blue and red arrows respectively), splicing out 5′leader and 3′ trailer respectively. (**B**) Here, each gRNA with target-specific sequence (labelled here as circles of different colors) and conserved gRNA sequence (blank rectangle) is fused to a tRNA coding sequence (rectangles with boxes), which is cleaved after transcription by RNaseP and RNaseZ to release mature tRNAs and gRNAs (with lines of same colors as the circles). These processed gRNAs direct Cas9 to the target site, which then causes a double-strand break (DSB), which is repaired by NHEJ or Homologous recombination (HR).

Transfer-RNAs (tRNAs) are a fundamental cellular component of all organisms, and their production and processing are mediated by RNA-processing systems. With this concept, Xie et al. (2015) developed an endogenous RNA-processing system to produce multiple gRNA from a single transcript (Figure 3). They have shown that a synthesized DNA fragment having tRNA–gRNA in a tandemly arrayed fashion can be proficiently processed into gRNAs having the desired 5′ targeting sequences, which precisely directed Cas9 protein for editing multiple chromosomal targets. The tRNA-processing system that includes RNaseZ and RNaseP, inherently present in a cell, precisely cleaves 5′ and 3′ ends of the tRNAs, thereby releasing individual gRNAs. By applying this strategy in rice plants, stably inherited mutations were readily achieved with up to 100% efficiency, and since tRNA processing machinery is nearly conserved in all the organisms, similar rates of mutation efficiency can be expected in a wide range of organisms. The tRNA-based multiple target editing is preferred over other methods due to several advantages, including the specificity of RNaseP and RNaseZ for tRNA. Only D-loop arm, acceptor stem and TψC-loop arm of tRNA are obligatory for the detection by RNase. [114]. The tRNAs also have an internal Pol III promoter site; therefore, tRNA sequences can also be tapped into as an enhancer system for Pol III. To explore whether the synthetic poly-tRNA-gRNA (PTG) DNA fragment would be transcribed, processed, and function as predicted, they synthesized PTG with the structures, tRNA-gRNA (PTG1 and PTG2) or tRNA-gRNA-tRNA (PTG1.1 and PTG2.1), and as a proof, the qRT-PCR analysis revealed that the level of PTG was 3 to 31 times higher than the simple sgRNA in rice protoplasts. Moreover, the full tRNA-gRNA transcripts were not detected by qRT-PCR, further confirming the efficient cleavage of gRNAs from the tRNA-gRNA transcripts by the tRNA processing system (Figure 3). Pol III promoters (e.g., U3p) similarly transcribe the PTGs as the sgRNA genes but, PTGs have an advantage as they are not obligated to begin with a specific nucleotide as is the case with sgRNAs. Therefore, the vectors that are currently used in CRISPR/Cas9 for the expression of sgRNAs can be used efficiently to express PTGs for the multiplexing approach (Figure 3). The PTG technology can be also be exercised for the improvement of induction of mutations simultaneously in multiple genomic loci, or for deletion of short fragments of chromosomes. For example, PTG could be used with Cas9 nickase to improve targeting fidelity [115,116,117] or with dCas9 transcriptional activator or repressor to manipulate multiple gene expression [118,119].

A noticeable limitation of the tRNA based multi-target-editing would be the reduction in efficiency of the PTG system if the number of gRNAs in the polycistron were increased beyond six [114]. This reduction in the efficiency of PTGs with a high number of gRNAs was probably due to the competition for Cas9 among gRNAs [114]. However, still, this system seems one of the most efficient systems to perform multi-target genome editing. The tRNA based genome editing approach has already been exploited in other crop plants such as tomato [120], maize [121], and wheat [122]. An increasing number of studies exploring the approach will help to understand the technique and its pros and cons.

### 5.2. Engineering Introns to Express sgRNAs

**Figure 4:**
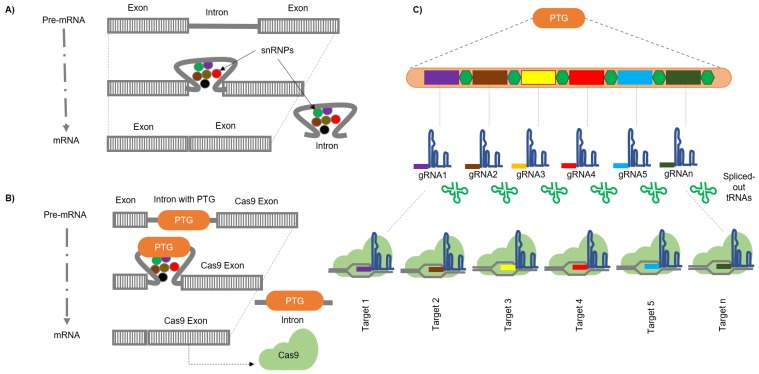
Schematic representation of multiplex genome editing by utilizing intron polycistronic transfer RNA-guide RNAs (inPTGs). Here, figure (**A**) depicts the regular small nuclear ribonucleoprotein (snRNP) mediated splicing mechanism. (**B**) Introns are engineered to code for fused polycistronic tRNA-gRNAs (PTGs). (**C**) PTGs are further processed to release individual gRNAs (shown here in different colors) via the tRNA processing machinery. Individual gRNAs can then go on to target their complementary loci in the genome.

Pol III and Pol II promoters express small nucleolar RNAs (snoRNAs) and microRNAs (miRNAs) respectively. The snoRNA and miRNA are mostly present in the introns of coding genes and processed by their biogenic pathway. Similarly, gRNAs can also be engineered from introns of Cas9 or Cpf1 by modifying the RNA processing machinery to precisely cut individual gRNAs without disrupting the standard splicing mechanism. Since introns are universal modules of a eukaryotic genome, they can be engineered to express gRNAs in virtually all the eukaryotes (Figure 4). Intron PTGs constructs (inPTGs) have comparable fragment deletion frequencies up to 30.9% with the PTG constructs. Full length and truncated introns have also been tested, and not much difference was found in the editing efficiency using these two, revealing that intron length has no profound influence in this method. The inPTGs can also be expressed by various Pol II promoters at different positions in the host genome efficiently, rendering inPTGs flexibility to enhance the editing efficiency further. The inPTGs have also been successfully applied in CRISPR/Cpf1 crRNAs system, and the gene editing efficiency of intronic crRNAs was almost two-fold higher than conventional U3p expressed crRNAs. In addition to the benefits of using Pol II as promoters for gRNAs with Pol III terminator, inPTGs allow loading of multiple gRNA-tRNA units as the introns are thousands of base pairs long. Moreover, the inPTG method allows the synchronization and balancing of Cas9/Cpf1 and gRNA/crRNA expression with robustness and flexibility, without introducing additional RNAs or ribonucleases, thus minimizing the risk of the potential toxicity of additional nucleases to the cell [123]. This intricate strategy developed by Ding et al. [123] to express multiple gRNAs from the introns of Cas9 gene, utilizing the endogenous tRNA system to splice out the gRNAs in Cas system or crRNA processing capability of Cpf1 nuclease, exhibits greater efficiency than standard Cpf1 vectors. Because of its simplicity, this approach can be used on a broader platform efficiently.

### 5.3. Csy4 Nuclease Mediated Multi-Targeting Genome Editing

**Figure 5:**
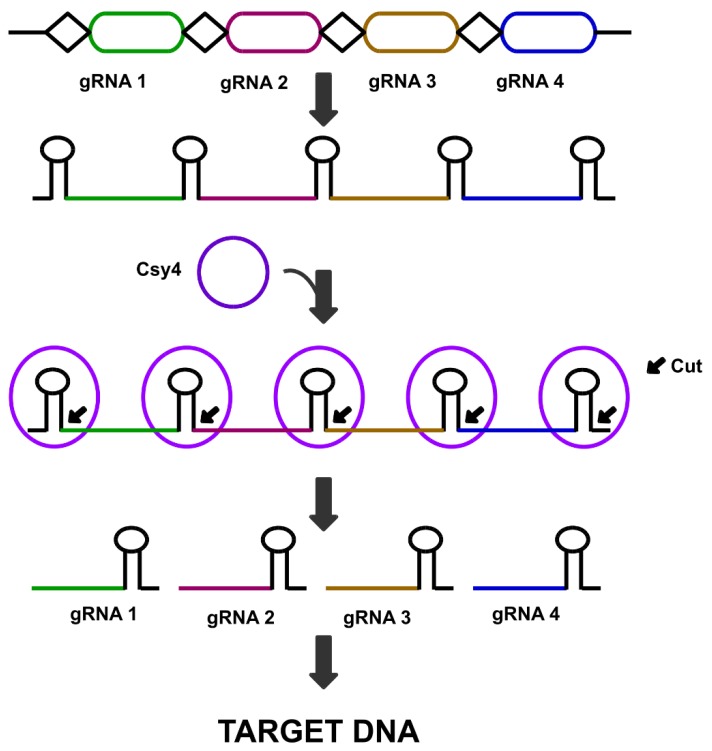
Multiplex gene editing using CRISPR system *Yersinia* (Csy4) endonuclease, shown here as blue circles. Csy4 restriction sites are cloned between each sgRNA, and Csy4 endonuclease gene is also cloned in the same vector. Expression of Csy4 endonuclease results in the separation of individual sgRNAs, which can then go on to target their respective sites.

The Csy4 endoribonuclease, from *Pseudomonas aeruginosa* has been effectively exploited to excise multiple gRNAs from synthetic polycistronic transcript [115]. Multiple gene expression was observed by designing gRNAs in a tandem array, each flanked by recognition sequences for Csy4 (Figure 5). Also, the bacterial origin of Csy4 makes it a suitable tool for building complex synthetic circuits without interfering with the endogenous RNA machinery of the host cell [124,125]. The successful functioning of Csy4 endonuclease has been shown in several, plants. The Csy4 endoribonuclease from *Pseudomonas aeruginosa* has a high substrate specificity towards a 28 nucleotides RNA stem-loop (5′-GTTCACTGCCGTATAGGCAGCTAAGAAA-3′) [126]. Once bound to the RNA stem-loop, Csy4 cleaves after the guanine at position 20, allowing to generate multiple RNA transcripts. The RNA processing ability of Csy4 can be applied for gene deletion and interference lucratively. The PTG and cys4 mediated multiplexing has also been successfully validated in tomato (*Solanum lycopersicum*), tobacco (*Nicotiana tabacum*), barley (*Hordeum vulgare*), wheat (*Triticum aestivum*), and *Medicago truncatula* [127].

### 5.4. Drosha-Based Multi-Targeting Genome Editing

**Figure 6:**
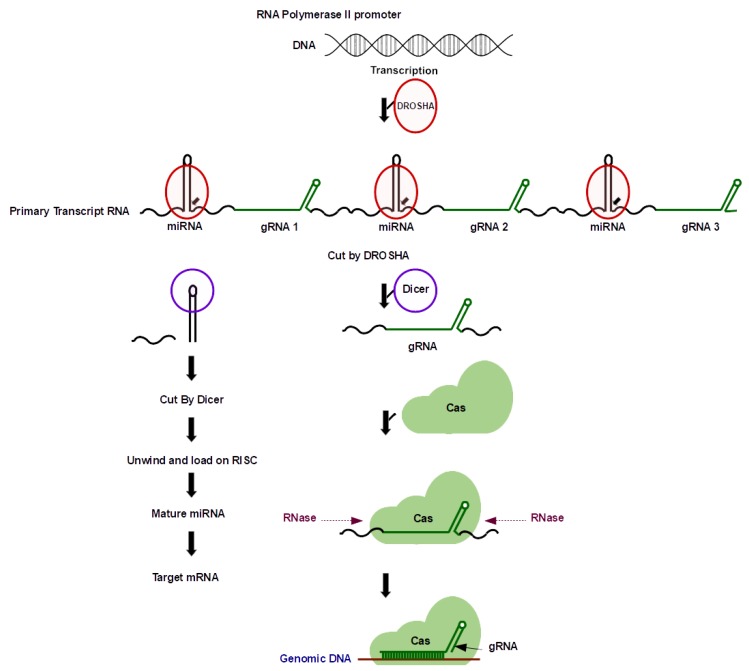
Drosha based approach for multiplex gene editing. In this system, gRNAs and miRNAs are cloned in a tandem array. Dicer cleaves the miRNA ends and thereby separating gRNAs also. The pathway on the left explains the general scheme for miRNA mediated mRNA targeting, and the one on right side explains miRNA-based gRNA multiplexing system. Abbreviations. miRNA: micro RNA; gRNA: guide RNA; RISC: RNA induced gene silencing complex; Cas9: CRISPR associated protein 9; RNase: ribonuclease; sgRNA: single guide RNA. Here, sgRNA and gRNA imply the same entity.

Drosha based multi-targeting genome editing approach is a multi-target genome editing approach in which tandem consecutively arranged miRNA (or shRNAs)-sgRNA genes are expressed under the control of a single polymerase II promoter. Generally, Pol III promoters are manipulated to express sgRNAs because of the lack of special structures such as 5′ cap, 3′ tail or introns, but they are inefficient because of short length and limited life of Pol III transcripts (Figure 6). Polymerase II transcribed sgRNAs are desired because of their ability to be expressed in a tissue-specific and flexible manner, but these have redundant nuclease activity because of the 5′ cap structure. This issue can be rectified by using miRNA-based strategy, using the microprocessor protein complex, comprised of Drosha, an RNase III enzyme, and its cofactor, DGCR8 or Pasha for the production of mature gRNAs and miRNAs [128]. Even though a highly robust approach, it is still relatively less preferred by the plant scientists.

## 6. Precision Editing/Base-Editing Approach

**Figure 7:**
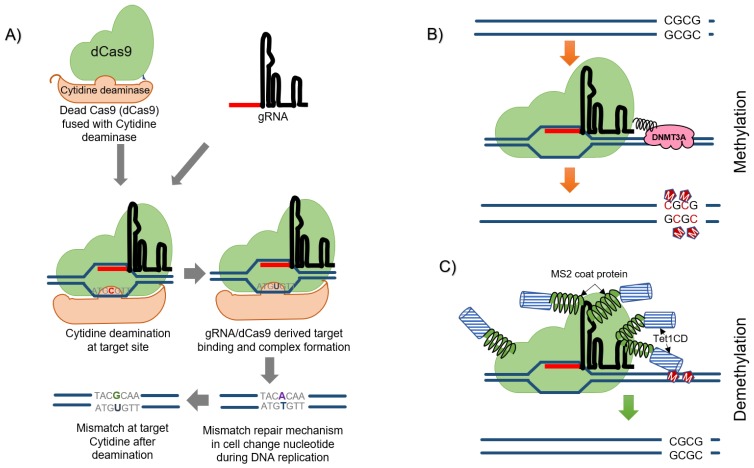
(**A**) Precision base editing by utilizing cytidine deaminase fused with dCas9. With the help of guide RNA (gRNA), Cas9 make complex at a specific target site and then the cytidine deaminase act on cysteine present on the opposite strand. The deamination process converts cysteine (C) to uracil (U) which later gets converted into adenine-thymine base-pair during DNA replication by the inbuilt mismatch repair mechanism. (**B**,**C**) CRISPR mediated methylome editing. Dead Cas (dCas) is fused to a DNA methyltransferase (DNMT3A in case of animals) or a demethylase, such as ten-eleven translocation dioxygenase (tet) in animals, which can be used to edit the epigenome.

Use of CRISPR/Cas approach is straight forward when it comes to generating knockouts, but precise base editing remains challenging because of the preference of NHEJ instead of HDR pathway for the repair of DSB in natural systems. Moreover, HDR also requires an oligonucleotide template (donor template) to be developed and transferred along-with CRISPR/Cas reagents into the cells, for target-specific recombination and gene repair. Methods have been developed to precisely edit bases in DNA without causing DSBs using CRISPR/Cas mechanism by using modified chimeric Cas protein, having a DNA recognition module attached to a catalytic domain with the ability of chemically modifying the bases. The dCas9 guided by a sgRNA is used most of the times in such a system, with reduced insertions and deletions (Figure 7) [129]. First base editor developed by Liu et al. in 2016 is APOBEC deaminase fused to dCas9 that converted cytidine (C) to uracil (U). The resulting U-G mismatches subsequently form U-A, and finally, the T-A base pairs [130]. BE2 is another base editor developed afterward that uses Uracil DNA Glycosylase, which inhibits the excision repair pathway and can be added to increase efficiency. Another system that resulted in an improvement of the technique is BE3, that uses Cas9 D10A nickase, is similar to activation-induced cytidine deaminase (AID) and results in a six-fold increase in the efficiency. The CRISPR-X and targeted AID-mediated mutagenesis use such a strategy to generate mutations at localized sites [131,132]. Adenine DNA deaminases have also been developed, which do not occur naturally.

Although initially applied in animal systems aspiring to reduce the off-target editing, this system has also been effectively used in plant system—including wheat, maize, rice, cotton, potato, and tomato [133,134,135,136,137]—and provides an opportunity to tweak the vital agronomic traits which are due to single base-pair mutations. Base editing in rice targeting *ALS* and *FTIP1e* generated double mutants resulting in resistance to two herbicides [134] and can be further used to improve the 14 identified agronomic traits in rice and flavor and fruit weight in tomato as well [138]. Even though rates of indel are significantly higher in plants as compared to that observed in mammalian species, the indel frequency is still lower than the traditional HDR based CRISPR/Cas9 [34,139].

The indels produced by wild type Cas9 are random and do not ensure knocking out of a gene, rather some indels may also enhance the function of the protein to be targeted. The BE3 systems have also been used for CRISPR-STOP and iSTOP for base editing mediated introduction of early non-sense codons [140,141]. CRISPR-STOP and iSTOP produce knockouts via early truncation of target locus, which is in contrast to CRISPR interference (CRISPRi). Specific codons—namely CGA (Arg), CAA (Gln), TGG (Trp), and CAG (Gln)—can be targeted in the coding strand using BE3 to create TGA (opal), TAG (amber), and TAA (ochre) stop codons by changing G to A or C to T. Billon et al.(2017), have developed an easy to use database to rapidly detect the induced STOP codons in the genome of eight eukaryotes, which can be very helpful for precise and effective genome editing [140].

## 7. CRISPR Mediated Manipulation of Gene Expression: Promoter Bashing

The CRISPR/Cas system can be used to manipulate the expression levels of a gene by using catalytically inactive dCas9 fused to a transcriptional regulator. Earlier experiments done with animal cells implied that fusion of dCas9 with an activator, like VP64, could activate the genes but 3-4 gRNAs targeting a single promoter is required to activate a gene efficiently [118,142,143,144]. Piatek et al. (2015) have already demonstrated the successful application of such a system in plants wherein they have used dCas9 combined with EDLL and transcription activator-like (TAL), which are plant-specific activators, and SRDX, which is a repressor, using sgRNAs specific for the *Pds* gene [145]. Lowder et al. 2015, also discovered that the dCas9-VP64 activated miR319 and *AtPAP1 (Production of anthocyanin pigment 1)* by up to 7-fold, and could also reverse gene silencing due to methylation in *AtFIS2 (Fertilization-independent seed 2)* gene in *Arabidopsis* changing the *AtFIS2* expression levels by up to 400-fold [146]. One of the milestone studies includes the generation of diverse cis-regulatory alleles in tomato by editing the promoters of genes involved in major productivity traits in tomato, including fruit size, plant architecture, inflorescence, and branching [147]. These results show that CRISPR/Cas can be a powerful tool for robust activation and repression of protein-coding as well as non-protein coding genes.

## 8. CRISPR Mediated Editing of Methylome

DNA methylation is a very important epigenetic modification that controls many biological processes and gene regulation during development. Post-translational methylation of histone proteins and modification of the DNA by methylation of cytosine residues are the most important approaches for epigenetic gene regulation in organisms. DNA methylation is the preferred mode for targeted regulation of gene expression as it is more stable and can result in long-term effects. DNA methylation maps with single-nucleotide resolution have been developed for animal cells and specific tissues [148,149]. The same has been done with model plants [150], and this information can be used to define the differentially methylated regions during different stages of development or in case of a disease manifestation. Exploring the function of each methylation remains a very daunting task. However, now this can be accomplished by utilizing the engineered nucleases (dCas or Cpf1) protein fused to various enzymes, as is done in targeted base editing, by permanent establishment or erasure of methylation from the targeted nucleotides. For efficiently studying or for developing disease resistance and for other purposes related to methylation, CRISPR/Cas system can be used for methylation of specific sites in the genome responsible for activation or deactivation of a particular gene by fusing DNA methyltransferases to dCas9. DNA demethylases can also be used in a similar manner. CRISPR/Cas mediated methylation and demethylation has already been achieved in the animal system [151]. In animals, DNMT3a/b is responsible for de novo methylation, DNMT1 is the most operative for the maintenance of methylation and TET (ten-eleven translocation) dioxygenase is used as a demethylase as shown in Figure 7. At least three classes of DNA methyltransferases are present in case of plants, namely MET1, chromomethylases (unique to plants) and the remaining class is very similar to DNMT3 of animals, responsible for de novo methylation. Since plant DNA methyltransferases are very similar to the animal DNA methyltransferases, methylome editing can thus be performed in plants in a very similar fashion to animals. Very recently, CRISPR mediated methylome editing has been reported in *Arabidopsis*, where a modified SunTag approach has been used along-with the previously characterized catalytic domain of *Nicotiana tabacum DOMAINS REARRANGED METHYLTRANSFERASE* (*DRM*) to act as a methylation effector, for the manipulation *FWA* and *SUPERMAN* genes. This work is also an insightful example of promoter bashing (using dCas9-VP64 fusion) and site-specific base editing [152]. However, epigenome editing using CRISPR/Cas has been applied in the plant kingdom very scarcely, but it certainly offers an extensive area of opportunities to the researchers working all over the world, especially for the improvement of crop plants.

## 9. Concerns about Off-Targeting Activities of Cas Protein

The off-target activity of Cas9 protein is a major concern as Cas protein can edit any DNA sequence having about five mismatches with its sgRNA [153,154], however, in most cases, Cas9/sgRNA cannot recognize a DNA sequence with mismatches greater than three with the sgRNA. Although the off-target activity observed in plants is at a superficial level, still there is a need to develop straightforward methods to overcome the pitfall of off-target effects to ensure specificity of editing by Cas9, increasing the number of targetable sites and fidelity of Cas9. Many features of the CRISPR/Cas9 reagents have been characterized regarding the off-targeting tendency of Cas9. Reports suggest that Cas9/sgRNA cannot recognize and edit a DNA site with mismatches within 10–12 bp of the PAM site, and the Cas9 protein has a higher affinity for NGG-PAM than for NAG-PAM [155]. Several strategies have been devised to overcome this obstacle of high off-target to on-target ratio. The easily adaptable feature is a lower concentration of the CRISPR/Cas reagents promote specificity of targeting [155]. Other strategies include: selecting target sites with a higher GC content (up to 70%) [156]; truncating sgRNAs at 3′ [157,158] or 5′-end [159] and adding extra GG at the 5′ end; using paired sgRNA Cas9 nickases; utilizing the FokI nuclease to guide Cas9 [115,160]. Potential off-target sites can also be identified using next-generation sequencing-based methods such as ChiP sequencing [161], digenome sequencing [162], and GUIDE-seq [163]. Many bioinformatics tools have also been developed, which can help to reduce the off-target effect. Cas9 variants with a D10A [116] or H804A modification which converts the Cas9 into a nickase or dCas9 fused to FokI nuclease domain [117], have been developed requiring two sgRNAs to target one site and thereby reducing the scope for off-targeting. Cas9 variants with 3-4 amino acid substitutions, which do not tolerate even a single mismatch has also been developed [164]. These Cas9 variants can be readily used to address the issue of off-target effects in CRISPR/Cas9 technology. Nevertheless, the off-target concern is not that serious in case of plants as the mutations can be easily segregated out by performing a couple of backcrosses.

## 10. Tools Available for Designing sgRNA and Detection of Potential Off-Target Sites

Selection of appropriate target sites is an imperative aspect for reducing the problem of off-target effects of Cas9 and for ensuring that the target site selected is in the coding region of the gene. Various bioinformatics tools are now available, which help in selecting appropriate target sites and designing the sgRNA accordingly, which virtually eliminate the need for applying the above said measures to reduce the off-target effects. Various other measures can also be kept in check, such as the GC content of the target site and restriction sites within the selected target. Some of these tools are given in Table 2.

## 11. Challenges for Plants Difficult to Transform

Although CRISPR/Cas is an unparalleled and pioneering technology, still there are some bottlenecks for its effective application in the area of crop improvement and translational research. One of those bottlenecks is efficacious delivery of transformation vectors into the correct host cells and further successful regeneration of plants. Plant transformation progresses through two consecutive steps: Transient transformation and stable transformation. Stable transformation being responsible for the production of edited plants having heritable mutations, of which the integrated transgene of nuclease can be segregated away to produce transgene-free plants.

*Agrobacterium-*mediated and biolistic transformation methods remain the most applied transformation methods, but they are incompetent for many crops with the main challenges being: (1) low incidence of stably transformed plants, (2) long tissue culture periods, (3) tissue damage due to biolistic transformation, (4) limitation of *Agrobacterium-*mediated transformation to very few genotypes in a species, (5) *Agrobacterium* induced browning and necrosis of tissues, (6) induction of somatic mutations, (7) difficulties in transforming monocot species using *Agrobacterium*, and (8) low quantities of DNA delivered via *Agrobacterium-*mediated transformation which are insufficient for efficient HDR. Also, most of the time, cells easily regenerated cannot be transformed or vice versa and meticulous optimization of tissue and cell culture media with appropriate growth regulators are required [179]. Therefore, alternate transformation strategies that are proficient, easy to use, labor-saving, and simplified are required. Some of the improved transformation strategies are given in the following section.

### Hand-In-Hand Improved Transformation Approaches

Some advancements in strategies used for plant transformation have already been made, and much work is simultaneously going on. One such strategy is to increase the frequency of HDR because efficient HDR requires high titers of nuclease and repair template to be delivered into the plant cell. Viral vectors are being developed to deliver large numbers of nucleases and repair templates aiming at increasing the frequency of HDR [180]. *Agrobacterium* species with a broader host range should also be developed. Many bacterial species other than *Agrobacterium*, capable of transforming diverse plant species have also been reported. These include *Sinorhizobium meliloti, Mesorhizobium loti, Rhizobium* sp. NGR234 and other *Rhizobium* species, and *Ensifer adhaerens* [181,182,183], of which at least *Sinorhizobium meliloti* has been shown to be capable of infecting both dicots and monocots [184]. Most of the microorganisms capable of transferring genetic material into plant cells use *Agrobacterium*-derived DNA transfer machinery, but a *Rhizobium* species has been identified recently which is capable of transferring DNA with its protein apparatus [185]. *Tobacco rattle virus (TRV)* based vectors used for virus-induced gene silencing (VIGS) have also been developed and demonstrated to work efficiently for the delivery of gRNA and Cas9 in *Nicotiana benthamiana* germline cells, bypassing the requirement for plant transformation, and can infect multiple plant species. *TRV* based system is also crucial because the viral RNA genome does not integrate into the plant DNA genome and thus is a desirable factor to generate transgene-free plants [186]. Recently, Lowe et al. (2016) developed a ‘genotype independent’ method to improve the transformation efficiencies in several recalcitrant monocot species including maize, sorghum, sugarcane, and rice. In this technology, they expressed developmental regulators Baby Boom and Wuschel2 to stimulate the proliferation of transformed cells and recover transgenic plants at higher frequencies. [187]. Many other novel methods—such as magnetofaction using iron nanoparticles for pollen transformation [188], agar-trap method for liverwort transformation [189], mesoporous silica nanoparticles [190,191], carbon nano-fibres [192], and fluorescently labeled starch nano-particles [193]—have been developed which majorly use particle bombardment but can be used on a very wide platform and with much higher efficiency. Of these advancements, pollen magnetofaction is particularly interesting in that it provides an opportunity to generate transformed seeds directly without regeneration, and is genotype independent as well as culture-free. This technique has been successfully applied in cotton, which is difficult to regenerate. The transgene was shown to integrate into the genome stably, effectively expressed, and transferred stably to the subsequent generations [188]. The use of regeneration boosters to help regenerate recalcitrant species seems to have tremendous applicability. Dependence on resistance marker gene for the selection of plants in the CRISPR/Cas application is tedious, and at least two generations must be screened to check the efficiency of the transgene. The use of fluorescent proteins as a selectable marker, in recent years, has emerged as a speedy method to identify transgene. In *Arabidopsis thaliana*, it has been successfully applied to check transgene expression [194]. However, due to the necessity of in vitro transformation protocol, it has not been tested in other species. Aliaga-Franco, Cunjin [195] applied the Golden Braid cloning system in Arabidopsis, rice, and tomato and used DsRed fluorescence as a marker that operates accurately in dry seeds and helps to select transgene-free dry seeds. This technique has also been applied in hexaploid wheat (*Triticum aestivum)* for the production of male-sterile lines by targeting *Ms1* (Male fertility gene) [196]. Using a marker-free system is another advancement that utilizes the competence of transformed and non-transformed entities to regenerate by using regeneration promoting factors such as cycD3 gene, cytokinin, and auxin-related genes [197]. Discovery of simplified protocols for plant transformation not requiring plant tissue culture, easily reproducible in multiple labs and not requiring much expertise is needed for the application of gene editing on a broad range of plant species.

## 12. DNA-Free Reagent Delivery Methods

CRISPR/Cas mediated genome-edited plants can be classified as non-genetically modified. However, the most frequently used methods for the delivery of constructs also lead to the insertion of transgene/s in the plant genome, therefore resulting in transgenics and a need to segregate the inserted genes in the following generation. However, this process is more troublesome if transgenes are inserted at multiple sites into the genome. Additionally, RNP mediated genome editing is desirable for clonally propagated plants. Hence, more novel technologies to altogether bypass the transgenic regulations are required. One such method is the production of Cas9 RNPs, which are composed of pre-integrated Cas9 nuclease and sgRNAs, and are equally efficient to generate genetic alterations as DNA-based transformation methods. In case of plants, RNP complex delivery is inhibited due to the presence of cell walls and thus is incorporated into naked plant protoplasts. RNPs have been successfully used in wheat [198], Petunia [199], lettuce, rice, Arabidopsis and tobacco [200], maize [201], and also in grapes and apple to improve biotic resistance. [81]. Svitashev et al. (2016) [201] have reported much higher mutation frequencies using RNPs for CRISPR/Cas than using plasmid-based approach. Efficient delivery and higher cleavage rate, along with the probability of bypassing the transgenic regulations, make RNPs a tool of choice.

## 13. Germline-Specific Gene Expression

A lot of early experiments using CRISPR/Cas equally involved rice and *Arabidopsis*, but the efficiency of transmission of mutations to subsequent generations was much higher in rice than in *Arabidopsis.* The higher efficiency of mutation inheritance was evident from the fact that in experiments evaluating knocking out effects of CRISPR/Cas involving *Pds*, the albino phenotype was transmitted to subsequent primary transgenic generation in rice [36]. However, *Arabidopsis* took several generations to obtain the homozygous mutants, with 1-bp deletions and chimeric mutations predominating and most of the mutations being in somatic cells [202]. This phenomenon is most likely due to different DSB repair capacity among different cell types. This prompted researchers to use a different cell or tissue-specific promoters to optimize gRNA and Cas nuclease expression, including germline-specific gene expression. Some examples of such promoters are given in Table 3. One of the disadvantages associated with germline-specific promoters is that there is no way to check at an early stage if sgRNA is being expressed well as no expression can be detected in vegetative tissues. Therefore, if the target sites are not edited in T_0_ plants then one might have to wait till T_2_ or even further generations. Still, such promoters can be used to increase the gene editing frequency in recalcitrant species and offer vast potential for technological improvement.

## 14. Future Prospects

CRISPR/Cas based genome editing emerged as a game-changer in recent years due to its enormous potential to make desired modifications in the genome and also for versatile diagnostic purposes. The technological advances are being implemented not only for generating knockouts but also knock-ins, and for activation as well as repression of gene expression. Because of its utility in a practical system, many advancements have already been achieved in a very short period after its discovery, that includes DNA free genome editing systems (RNPs), multiple Cas9 variants, many multi-gene targeting approaches, precise base editing, and measures to increase the frequency of HDR. Advancements in the CRISPR/Cas related bioinformatics tools have also led to the elevated use of this powerful genome editing technique. However, the challenge of providing the public with sufficient knowledge regarding CRISPR/Cas mediated method of modifying crops cannot be discounted, as this is essential to take laboratory research to the masses, to provide agriculture with a sustainable future. In a case, it is essential to have laws that clearly mark a line between the gene-edited plants having foreign DNA and gene-edited plants without any foreign DNA, rendering the latter subject to be bypassed for any regulations, making the utilization of such plants very trouble-free. As compared to the commercial release of transgene-free genome-edited plants having a novel mutation, genome-edited plants with mutation precisely the same which exist with natural variation will be more relaxed, more particularly in countries where policies are not in the favor. In this regard, the precise genome editing approaches look promising. The gap between lab to the field is continuously widening because of the regulatory policies which are not yet well defined. Since gene editing may lead to genome modifications which occur in nature and the results of genome modifications implemented by the ‘traditional’ methods and genome editing cannot be distinguished in some cases, and, therefore, special regulation of the technology is unreasonable. Another opinion states that the legislator should concentrate on the specific characteristics of the final product rather than on the production method to identify the possible risk in any case. At the same time, one must not forget about the obligation for proper assessment and management of risks, regulation of the gene drive issue, and essential precautions for the use of this technology in the field of reproduction and human genetics. Apart from these issues, significant efforts are required to improve the transformation techniques which is not yet optimized for several crop species and highlighted as a bottleneck to explore recent advancement efficiently. The CRISPR/Cas based genome editing is still limited to sophisticated molecular biology lab which mostly focuses on the fundamental biological question. In contrast, crop breeders still need to make significant efforts to implement technological advances in crop improvement programs. Pay-per-use basis, public as well as private facilities providing service for construct development, transformation, and evaluation of genome-edited plants, will be game-changer to take the advancements from lab to field by facilitating crop breeder with most efficient genome editing tools. The information provided here will be helpful to understand the recent technological advances, knowledge gaps, problems with technological adaptations which are required for the efficient utilization of genome editing tools for crop improvement.

## Figures and Tables

**Table 1:** Significant studies demonstrating versatile use of genome editing approaches in plants

Mechanism of Action.	Plant Species	Target	Protein	Type of Mutation	Promoter	Vector	Reference
Knockout	Apple	*PDS*	pcoCas9 fused to GFBSD2	InDels	NA	pEgP226-2A-gfbsd2	[39]
Knockout	*Arabidopsis*	*BRI1, GAI, JAZ1*	hSpCas9	InDels	NA	NA	[40]
Knockout	Banana	*PDS*	Cas9	InDels resulting in early stop codon	NA	pRGEB31	[41]
Knockout	Rice	*OsROC5*, *OsDEP1*	*Arabidopsis* codon-optimized Cas9	Small deletions of up to 10 base pairs.	NA	pZHY988	[42]
Knock-in	Rice	*OsPDS*	SpCas9	Successful insertion of Oligo with KpnI+EcoRI sites resulting in gene disruption.	ZmUbi	pEASY-Blunt vector	[36]
Knock-in	Rice	*Chlorophyllide-a oxygenase gene of rice (CAO1)*	Monocot optimized FnCpf1	Heritable targeted insertion of repair template having hygromcin resistance.	ZmUbi	pUC19 backbone	[43]
Knock-in	Rice	*ALS*	pcoLbCpf1	Targeted ALS gene replacement resulting in Herbicide resistant plants.	NA	pCXUN-LbCpf1	[44]
Knock-in	Arabidopsis	*GLABRA2 (GL2); ROS1; DME*	hSpCas9	GFP-DME; DME-GFP; ROS1-GFP; ROS1-luc fusions were generated	AtU6	pCambia1300; pCambia3301	[45]
Knock-in	Maize	*ALS2; LIG*	SpCas9	Targeted insertion of MoPAT gene in LIG locus by upto 83% and mutation of ALS2 with by HDR with two oligos.	Ubi	pUC19; pSB11	[46]

**Abbreviations:** AtU6: *Arabidopsis thaliana* U6 snoRNA promoter; Cas9: CRISPR associated protein 9; Cpf1: CRISPR from *Prevotella* and *Francisella 1;* FnCpf1: *Franciella novocida* Cpf1; hSpCas9: human codon-optimized *Streptococcus pyogenes* Cas9; InDels: Insertion or deletion mutations; NA: Not Available; pcoCas9: plant codon-optimized Cas9; pcoLbCpf1: plant codon-optimized *Lachnospiraceae bacterium* Cpf1; SpCas9: *Streptococcus pyogenes* Cas9; Ubi: ubiquitin promoter: ZmUbi: *Zea mays* ubiquitin, NA: not available.

**Table 2:** CRISPR associated tools

Tool	Specialization	Specific Feature	Restriction Site Compatibility	Off-Target Analysis	Website	Reference
**ZiFiT**	Animal	Identifies all potential off-target sites. Although originally designed for ZFNs but now also applicable to CRISPR/Cas.	Absent	Present	http://zifit.partners.org/ZiFiT/	[165]
**CRISPR Direct**	Animal and plant	Results in the output of a table of candidate sites with their sequences, main sequence features, the number of unique matches in the genome, Tm, GC%, position of target site in sequence and ”12-mer + PAM” match numbers. Accepts accession number, genome location, and nucleotide sequence as an input. Can also check species specificity of sequence and mismatches, indels with the specific genome etc.	Present	Present	http://crispr.dbcls.jp/	[166]
**E-CRISP website**	Animal, bacteria, fungi, and plant	CRISPR/Cas9 targeting for different protein tagging experiments. User can search and import ENSEMBLID within the E-CRISP site. Ranks gRNAs according to on-target specificity and number of off-targets, can adjust stringency, design based on specific purpose. Gives an SAE score (S—specificity, A—annotation, E—efficiency) to each target site.	Absent	Present	http://www.e-crisp.org/E-CRISP/	[167]
**CRISPRSeek**	Animal, fungi, bacteria, and plant included in Bio String based genome data packages.	Optionally filters gRNAs without restriction enzyme site, or without paired guide RNAs and fetches gRNA flanking sequences as well and indicates whether the target and off-targets are located in exon region or not. Genome-wide search for scores, ranks. An offline tool and comes as a software package.	Present	Present	https://bioconductor.org/packages/release/bioc/html/CRISPRseek.html	[168]
**flyCRISPR Optimal Target Finder**	Animal; invertebrates	Uses user specified sequences rather than target genomes. Can be used with varying stringency. User can directly enter the target sites to evaluate them and confirm the genomic location, strand, and species specificity of the target.	Absent	Present.	http://targetfinder.flycrispr.neuro.brown.edu/	[169]
**CHOPCHOP**	Animal and plant	Supports Cas9, Cas9 nickase, Cas13, TALEN, and Cpf1 and the purpose (knock-out, knock-in, activation, repression, or nanopore enrichment) can be specified. Target location (5′ or 3′ UTR, coding region, specific exons, promoter or splice sites), GC%, self-complementarity, length of flanking sequences to be displayed, restriction enzyme company preference as well as size of restriction enzyme binding site can be specified. Also designs primer options for the selected target for user to choose from. User can also add new species to the database.	Absent	Present	http://chopchop.cbu.uib.no/	[170]
**CRISPR-Multitarget**	Animals and plants	Input is in the form of a sequence or gene or transcript identifiers based on the genomes available in the software and works for Cas nickase as well. Designed to work with constitutive as well as alternative exons present in particular transcripts. User can specify 5′ dinucleotide, target length, PAM orientation. Versatile and can accommodate almost any possible target specificity of CRISPR/Cas system.	Absent.	CRISPR-Multitargeter gives links to GT-scan and Cas-OFFinder to perform off target analysis.	http://multicrispr.net/	[171]
**sgRNAcas9**	All organisms	An offline tool. Truncated sgRNAs can also be designed. Extracts nucleotide sequences flanking the target cleavage sites to design PCR primers for the validation of mutations by T7E1 cleavage assay.	-NA-	Present	https://sourceforge.net/projects/sgrnacas9/;www.biootools.com	[172]
**CRISPR-P**	Plants	Scores all the possible CRISPR Target sites, with option to customize PAM and on-target score. Input should be in FASTA format or gene or transcript identifiers. sgRNA length, snoRNA promoter, PAM sequence specific for a variety of Cas and Cpf nucleases. Tells about locus, gene, GC content, secondary structure of sgRNA and position of the sequence in the chromosome.	Present	Present	http://cbi.hzau.edu.cn/cgi-bin/CRISPR	[173]
**SSFinder**	-NA-	Freeware, easy to edit, and low memory demand tool compatible with many commonly used operating systems.	-NA-	-NA-	https://omictools.com/ssfinder-tool	[174]
**GT-Scan**	Animals and plants	Calculates GC%, number of mismatches as well as exact matches. User can set high specificity mismatch limit. Displays the position and location of the target in the genomic database, and the strand on which the target is present as well.	Absent	Present	http://gt-scan.braembl.org.au/gt-scan/	[175]
**CRISPR gRNA Design Tool (DNA2.0 design tool)**	Animals and plants *(Arabidopsis)*	User can check the specificity of self-designed gRNAs in specific genomes. Limited species number. User can choose between wild type Cas9 or nickase. Allows you to visualize the position for your gRNA relative to the splice variants and any overlapping genes.	Absent	Present	https://www.atum.bio/eCommerce/cas9/input	
**CCTop**	Animals and Plants	Evaluates target sites within the input sequence against a genome database. Displays the off-target site details (coordinates, gene ID, distance, etc.).	Absent	Present	https://crispr.cos.uni-heidelberg.de/	[176]
**Cas-OFFinder**	Animals, plants and others (fungi, bacteria, virus)	Online or downloadable program. Searches potential off-target sites for a variety of CRISPR/Cas systems. In addition to base mismatches, DNA or RNA bulges are included in the search and results can also be filtered.	-NA-	Present	http://www.rgenome.net/cas-offinder/	[177]
**Breaking-Cas**	All eukaryotic genomes present in ENSEMBL. (protists, fungi, bacteria, plants and animals)	Accepts nucleotide FASTA as input. Cas9 and Cpf1 variants, as well as customized PAM specificity, PAM position (5′or 3′), sgRNA length. Most extensive number of genomes are accessible.	Absent	Present	http://bioinfogp.cnb.csic.es/tools/breakingcas	[178]

**Abbreviations:** CRISPR—Clustered Regularly Interspaced Short Palindromic Repeats. Cas—CRISPR Associated protein. GT—gene target. NA—Not available. PAM—Protospacer adjacent motif. RFN—RNA-guided FokI Nuclease. sgRNA—single guide RNA. Tm—Melting temperature. ZiFiT- Zinc Finger Targeter.

**Table 3:** Tissue-specific promoters which can be used in CRISPR/Cas9 technology

S.No.	Specificity	Promoter	Reference
1.	Egg cell specific	*EC1.2* promoter	[203]
2.	Germ-line-specific	*SPOROCYTELESS*	[204]
3.	Meiocyte-specific promoter	*AtDMC1*	[205]
4.	Pollen-specific promoter	*Lat52*	[204]
5.	Egg cell- and early embryo-specific promoter	*DD45*	[204]
6.	Dividing tissue specific	*INCURVATA2*	[206]
7.	Cell-division specific	*YAO*	[207]
